# Triple Clavicle Injury: A Fracture of the Middle Third Associated With Acromioclavicular Dislocation and Sternoclavicular Subluxation

**DOI:** 10.7759/cureus.74828

**Published:** 2024-11-30

**Authors:** Gustavo G Ribeiro, Elisa G Figueiredo, Thiago A Souza, José Carlos S Vilela

**Affiliations:** 1 Orthopedics and Traumatology, Hospital Francisco José Neves - Unimed Belo Horizonte (BH), Belo Horizonte, BRA

**Keywords:** acromioclavicular joint dislocation, bipolar clavicle dislocation, sternoclavicular joint injury, triple clavicle injury, tripolar clavicle lesion

## Abstract

In this report, we present a case of a triple clavicle injury, acromioclavicular joint (ACJ) dislocation, a middle third clavicle fracture, and a sternoclavicular joint (SCJ) subluxation, and describe its successful surgical treatment. A 49-year-old female patient sustained a 3 m fall, resulting in direct trauma to her left shoulder. Initial radiographs and computed tomography (CT) scans revealed a displaced middle third clavicle fracture, a high-grade ACJ dislocation, and a posterior SCJ subluxation. Surgical intervention was proposed due to the severity of the ACJ dislocation. The patient underwent open reduction and internal fixation (ORIF) of the clavicle fracture with an anatomical locking plate (EVOS Clavicle Plate®; Smith & Nephew, Andover, MA) and ACJ stabilization using an Endobutton® (Smith & Nephew, Andover, MA) along with the transposition of the coracoacromial ligament. The SCJ subluxation was reduced indirectly. She returned to work after four weeks and, after six months, demonstrated excellent functional recovery achieving a full, pain-free range of motion. Outcome scores were favorable (Constant Shoulder Score: 73 bilaterally; University of California, Los Angeles {UCLA} Shoulder Rating Scale: 33). Follow-up radiographs at nine months demonstrated stable fixation with satisfactory anatomical alignment and fracture healing.

Given the absence of specific guidelines for managing this combination, we based our method on established protocols for isolated injuries. Our patient’s favorable outcome supports the effectiveness of this strategy and highlights the potential for successful functional recovery with careful management. This case underscores the importance of a high index of suspicion for concomitant ACJ and SCJ injuries in patients with midshaft clavicle fractures. This triad requires an individualized treatment plan for optimal outcomes, and future studies should focus on further documenting and evaluating treatment strategies for this rare injury pattern.

## Introduction

Acromioclavicular joint dislocation (ACD) and clavicle fractures are, individually, among the most common traumatic shoulder injuries. When combined, the fracture usually involves the lateral third of the clavicle. Only a few cases of ACD associated with fractures of the ipsilateral middle third of the clavicle have been reported in the English-language literature. Reports of a triple injury involving ACD, middle third fracture, and sternoclavicular (SC) injury are even more uncommon, with only one case described to date [1]. Here, we present a case of this uncommon association, aiming to contribute to future investigations.

## Case presentation

A 49-year-old right-handed female patient, employed as a gas station attendant, with no regular engagement in sports or recreational activities, was evaluated in an outpatient clinic four days following a 3 m fall into a ditch that resulted in direct trauma to her left shoulder. Her medical history includes systemic arterial hypertension (SAH). She had initially been assessed in an emergency care unit immediately after the accident, where she received analgesia and immobilization with a Velpeau sling and was subsequently referred to our outpatient service for further evaluation.

On the examination of the left shoulder, the skin was intact, with a visible prominence over the middle third of the clavicle. The patient exhibited intense pain upon clavicle palpation, tenderness over the sternoclavicular (SC) joint, and pain with an anatomical alteration of the acromioclavicular (AC) joint. The neurovascular examination of the affected limb was unremarkable. Additionally, a non-displaced fracture of the right fibula was observed, which was managed conservatively by instructing the patient to avoid weight-bearing on the right lower limb. No complaints or evident injuries were noted in other areas or systems.

**Figure 1:**
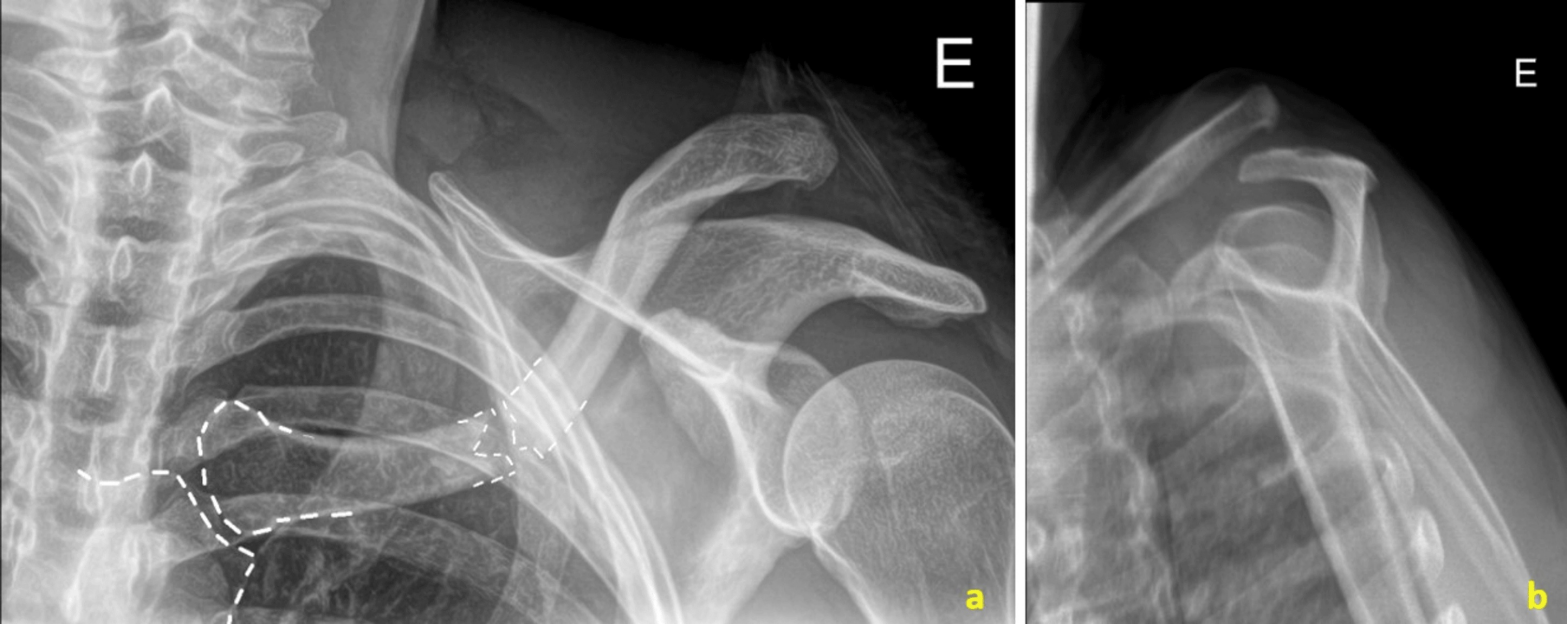
Radiographs of the patient at the initial evaluation in the emergency department. (a) AP view of the left clavicle with a dotted line highlighting the middle third clavicle fracture and the sternoclavicular joint. A high-grade ipsilateral acromioclavicular dislocation is visible. (b) Lateral scapular view illustrating the high-grade acromioclavicular dislocation.

**Figure 2:**
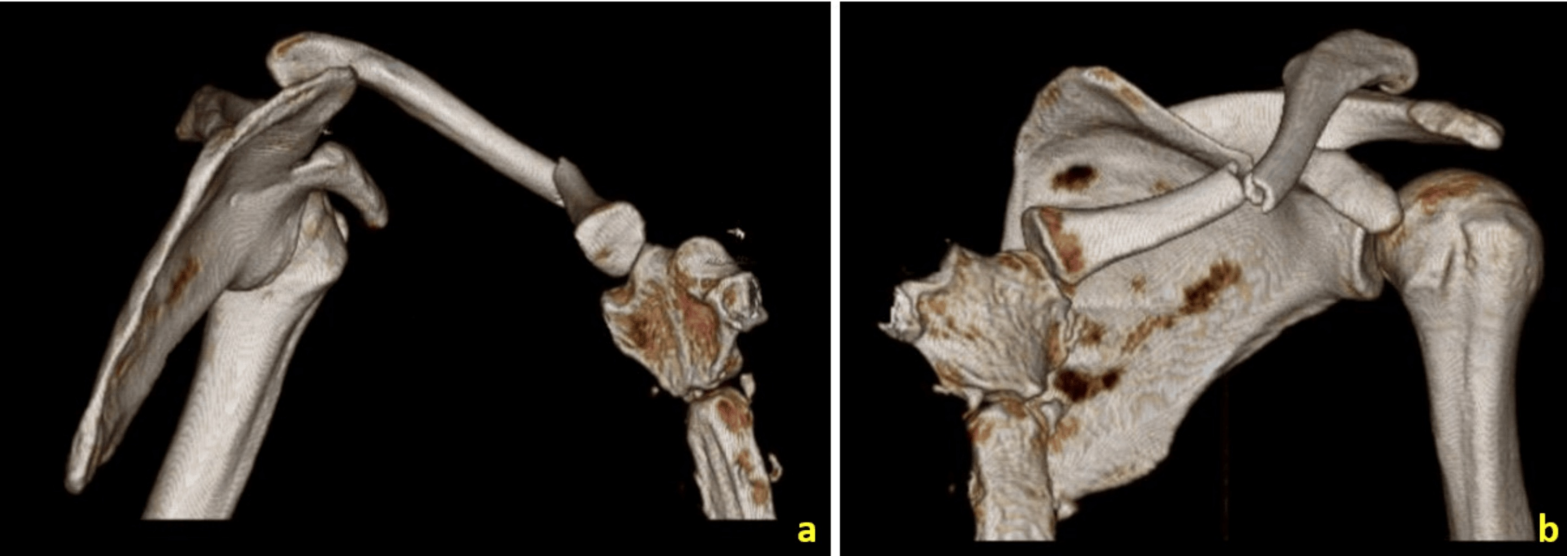
Computed tomography with a three-dimensional (3D) reconstruction of the left shoulder girdle. (a) Medial view showing posterior sternoclavicular subluxation. (b) Anteromedial view showing the displaced fracture with anterior angulation and high-grade acromioclavicular dislocation.

The initial radiographic examination revealed a displaced fracture of the middle third of the clavicle (Robinson type 2A2), a type V ipsilateral AC joint (ACJ) dislocation according to the Rockwood system, and suggested an SC joint (SCJ) dislocation (Figure 1) [2,3]. Computed tomography (CT) with three-dimensional reconstruction confirmed these findings, showing an anterior displacement of the distal fragment and anterior apex of the fracture. There was a complete loss of AC joint congruency, with a posterosuperior displacement of the clavicle. The CT scan also confirmed the posterior subluxation of the SC joint, which can be classified as type II by Allman, indicating a moderate sprain of the SC ligaments that presents as subluxation (Figure 2) [4].

Surgical treatment was jointly decided upon, taking into account the patient’s needs and the well-established indication for the surgical management of type V acromioclavicular dislocations (ACD) [5]. We deemed open reduction and internal fixation (ORIF) of the middle third clavicle fracture beneficial to perform in the same surgical session, based on limited specific literature and considering relative indications for surgical fixation, despite the concurrent ACD and/or SC joint injuries, as will be discussed in this paper [6].

The patient underwent surgery 10 days post-trauma, in a “beach chair” position under general anesthesia, with the left upper limb hanging freely. A longitudinal incision was made over the topography of the lateral two-thirds of the left clavicle, extending laterally for AC joint exposure. After layer-by-layer dissection, the imaging findings were confirmed. The fracture was anatomically reduced and temporarily fixed with a Kirschner wire. Definitive fixation was achieved with an anatomical locking plate (EVOS Clavicle Plate®, Smith & Nephew, Andover, MA), using three bicortical 3.5 mm locking screws in each fragment. The lateral end of the plate was shortened (two holes) to properly fit the patient’s anatomy, resulting in a plate with seven holes. The Kirschner wire was removed after plate application.

**Figure 3:**
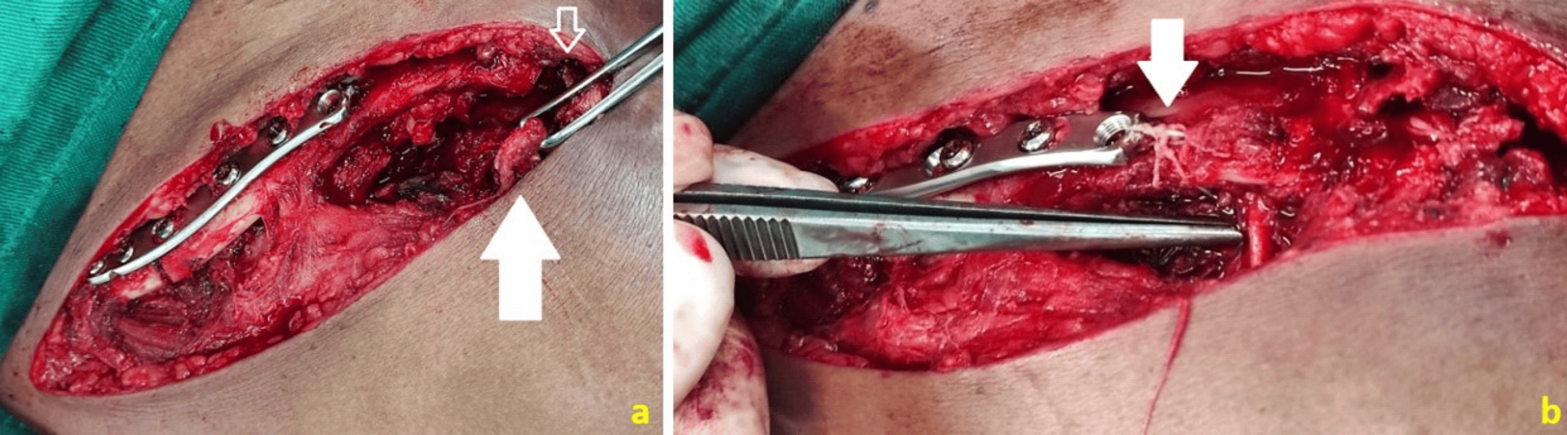
Anterosuperior view of the surgical site after open reduction and internal fixation (ORIF) of the middle third fracture, highlighting the acromioclavicular (AC) dislocation (open arrow) and the coracoacromial ligament resected from its acromial insertion (solid arrow) (a). At the conclusion of the osteosynthesis and AC fixation, the transposed coracoacromial ligament (tip of the surgical instrument) is noted to be fixed to the clavicle with a suture loop through a bony tunnel (solid arrow) (b).

The lateral end of the clavicle was displaced superiorly, with an increased acromioclavicular (AC) space observed following fracture fixation (Figure 3). This finding indicated a complete rupture of the coracoclavicular (CC) and acromioclavicular ligaments, along with the significant instability of the lateral fragment. A manual reduction was performed, followed by coracoclavicular fixation utilizing a single Endobutton® (Smith & Nephew, Andover, MA) through a bony tunnel in the coracoid and a suture loop through a bony tunnel in the lateral third of the clavicle with two number 5 Ethibond® (Ethicon, Inc., Somerville, NJ) braided polyester sutures.

Subsequently, a double AC loop was applied using two additional number 5 Ethibond® sutures, placed through two bony tunnels in the lateral end of the clavicle and two in the apex of the acromion, effectively restoring the bone anatomy. For the repair of the AC joint, the coracoacromial ligament was transferred, resected from its insertion on the acromion, and fixed to the lateral end of the clavicle via a single bony tunnel. Stability was further reinforced with a number 5 Ethibond® suture tied to the plate, enhancing vertical stability through a nonanatomical reconstruction of the CC ligaments, similar to the Weaver and Dunn technique [7].

**Figure 4:**
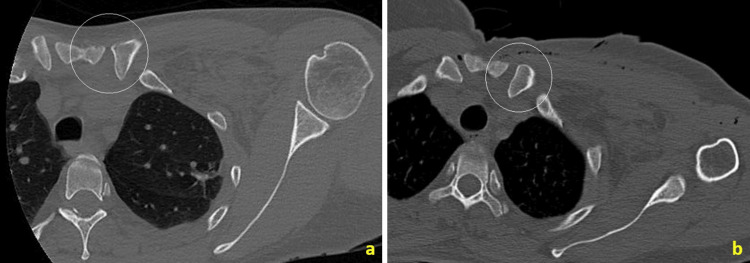
Axial cuts of the computed tomography scan in bone window settings at the level of the sternoclavicular (SC) joint in the preoperative (a) and immediate postoperative (b) phases. The left SC joint is highlighted by the white circles.

Posterior subluxations and mild to moderate sprains of the SC joint are successfully treated non-surgically, and closed reduction of these injuries can be attempted [8]. The patient underwent a follow-up CT scan after recovery from anesthesia, during the same hospitalization, to evaluate the SC joint. A satisfactory reduction of the subluxation was observed (Figure 4). Intraoperative tomography could have been an option to assess SC reduction after addressing the associated injuries; however, our center does not have such technology. The patient was discharged the following day, wearing a Velpeau sling on the left upper limb, and in good condition.

In the second postoperative week, the patient reported mild intermittent pain with no additional complaints. She was advised to discontinue the use of the sling and was referred for physical rehabilitation. A total of 20 physical therapy sessions were completed over the course of four weeks, after which she resumed her work activities.

**Figure 5:**
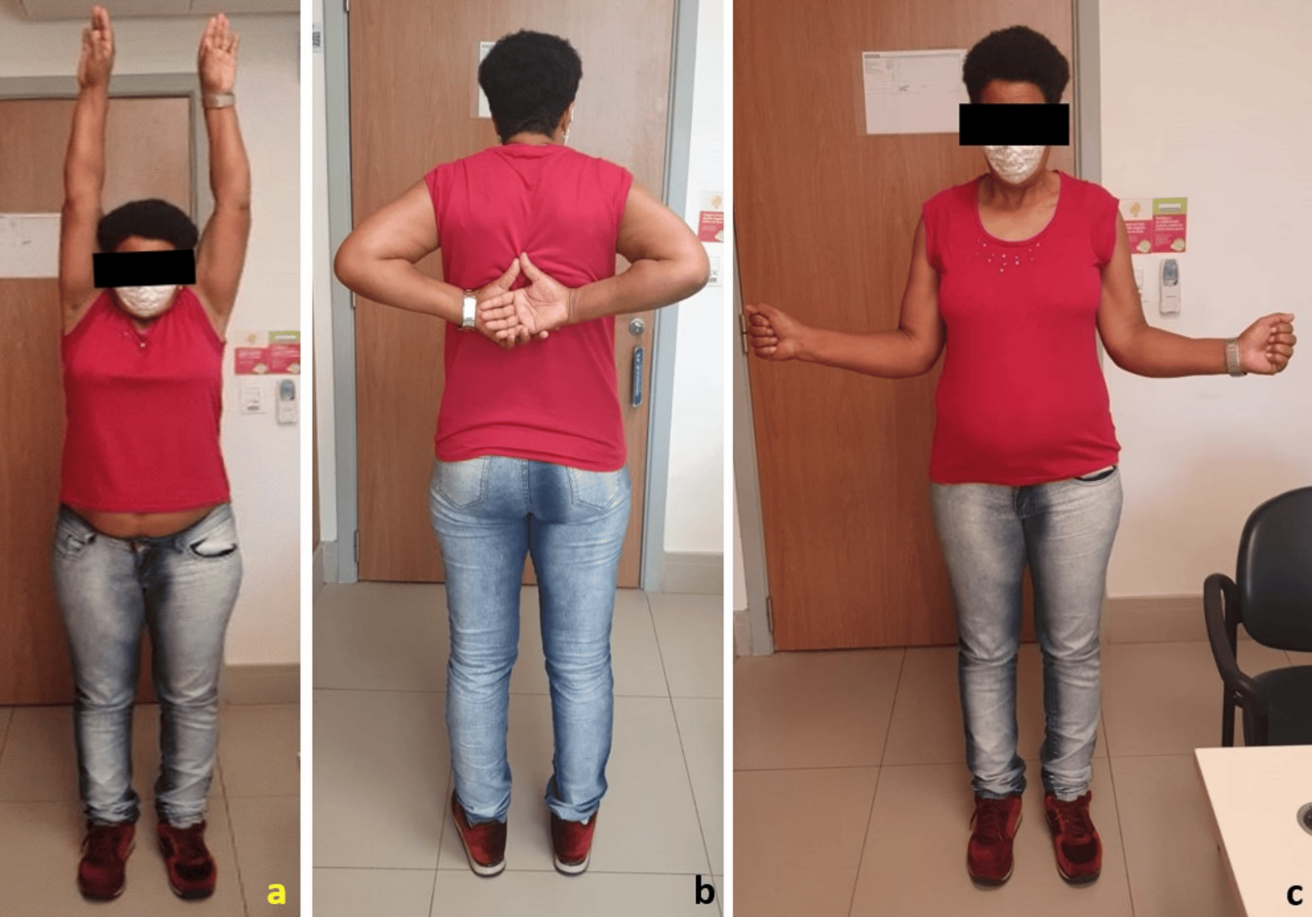
Assessment of the patient at the six-month postoperative follow-up, demonstrating a restored active range of motion symmetrically: abduction (a), internal rotation (b), and external rotation (c).

**Figure 6:**
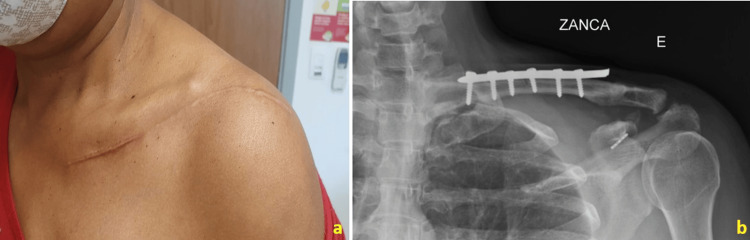
(a) Ectoscopic assessment of the patient at the six-month postoperative follow-up, demonstrating the postoperative appearance of the surgical incision with complete healing and no complications. (b) Radiographic control in the Zanca view of the left clavicle at nine months.

At the end of the six months of follow-up, the patient reported no spontaneous or provoked pain and had regained full range of motion in the left upper limb without limitations (Figures 5, 6). In the evaluation using the Constant Shoulder Score, she scored 73 on both sides, which is classified as an excellent result. On the University of California, Los Angeles (UCLA) Shoulder Rating Scale, she achieved 33 points, indicating a good/excellent outcome. At the nine-month follow-up, the patient maintained good objective results, with no other complaints or reports of complications, having fully regained her pre-trauma functions. On examination, the surgical wound was healed, and there was a slight prominence of the fixation material but no related complaints. She exhibited a wide and pain-free range of motion, with no restrictions. Radiographic control showed satisfactory reduction and proper positioning of the fixation material (Figure 6).

## Discussion

Clavicle fractures in isolation are common, accounting for 2%-10% of all fractures, and are more prevalent in young men under 20 years of age, representing up to 44% of shoulder girdle injuries [6]. They can be classified using the method proposed by Robinson, based on the location, complexity, and displacement of the fragments [2]. Acromioclavicular joint injuries are also common, constituting 40%-50% of shoulder injuries in athletes [5]. These injuries can be classified according to the anatomical severity of the injury using the Rockwood system, based on the original work of Tossy et al. [9]. This classification groups the injuries into six types (I-VI), with increasing degrees of severity [3,9].

Injuries of the sternoclavicular joint, on the other hand, are rare, representing less than 3% of all shoulder girdle injuries. They may present as sprains, subluxations, or dislocations [4,8,10]. The presence of all three entities ipsilaterally is extremely rare, with no available incidence data. In our review of the English literature, we found only one similar report published by Elliott in 1986. This author described a case of a comminuted fracture of the middle third associated with the anterior subluxation of the sternoclavicular joint and acromioclavicular subluxation (type II), which progressed satisfactorily with conservative treatment. Six months later, the patient had resumed sports activities [1]. The association of two of these injuries, namely, a middle third clavicle fracture and acromioclavicular dislocation, is also uncommon. In a review of the English literature, we found 16 case reports, recently revised and published by Schots et al. Notably, there is a variety of treatment options available, all reported to have good outcomes [11].

The three identified cases treated conservatively are a type II acromioclavicular dislocation, treated clinically with a good outcome after one year; a type III acromioclavicular dislocation, which showed a clinically satisfactory result despite persistent AC displacement noted on radiographic evaluation; and a type VI acromioclavicular dislocation associated with an incomplete greenstick fracture, which demonstrated satisfactory outcomes with conservative treatment [11]. Five of the reported cases were treated with a surgical fixation of the acromioclavicular joint combined with the conservative management of the fracture. This includes three type IV acromioclavicular dislocations reported by Wurtz et al. (1992) [12], one case of type IV acromioclavicular dislocation reported by Wisniewski (2004) [13], and the case reported by Lancourt (1990) [14], which did not specify the classification of the acromioclavicular dislocation according to the Rockwood system, but the images presented in the original work are consistent with a type V acromioclavicular dislocation.

Four of the cases were treated with open reduction and internal fixation (ORIF) of the fracture, along with the stabilization of the acromioclavicular dislocation using a hook plate, as presented by Schots et al. [11] and Wijdicks et al. [15]. These cases required reoperation for the removal of the hook plate, with all patients achieving satisfactory outcomes [11,15]. Beytemür et al. employed the same technique in their report but did not proceed with the removal of the hook plate, as the patient had no complaints [16]. Solooki and Azad reported a case in which ORIF of the fracture was performed along with the stabilization of the acromioclavicular dislocation using two Bosworth screws, one of which was placed through the plate [17]. Psarakis et al. described treatment with ORIF and coracoclavicular stabilization using the TightRope® system [18]. Yeh et al. reported the treatment with ORIF and acromioclavicular stabilization using an autologous semitendinosus graft [19]. Both groups reported good results without the need for reoperation [17-19].

Despite the seemingly rare association between midshaft clavicle fractures and acromioclavicular dislocations, as indicated by the limited case reports in the literature, this condition may be more common than previously thought. Ottomeyer et al. conducted an interesting study with a retrospective analysis of a prospectively collected database. Among 383 patients with midshaft fractures, 26 (6.8%) were found to have ipsilateral AC injuries. They noted no statistically significant difference in incidence between those who received conservative and surgical treatment. The injuries identified were 69% Rockwood type II (18/26), 27% type III (7/26), and 3.8% type V (1/26). Due to the retrospective nature of the study, the treatments provided or the outcomes were not evaluated. The authors were unsuccessful in demonstrating the risk factors for this association. They suggest a thorough evaluation of the AC joint in patients with midshaft clavicle fractures [20].

There are no specific guidelines or comparative studies available regarding the treatment of the associated injuries presented in our report. Therefore, we proceeded with the well-established treatment of isolated injuries. The surgical approach for type V AC dislocation aligns closely with the current literature [5]. Various techniques have been described, including AC repair with pins, plates, and screws; the transfer of the coracoacromial ligament; and CC reconstruction, with or without the excision of the lateral third of the clavicle [5,21]. We opted for AC stabilization using a suture loop and CC stabilization through the transfer of the coracoacromial ligament.

In the presence of soft tissue injuries at both ends of the clavicle, significant instability, a fragment displacement greater than 100%, the risk of exposure, patient preference, and the previously indicated lateral surgical approach, we deemed surgical treatment appropriate. The use of an anatomical locking plate aligns with the literature, our practice, and the availability of fracture fixation materials [6]. Despite the anticipated anatomical adequacy of the EVOS Clavicle Plate®, it was necessary to use a longer plate, consisting of nine holes that were sectioned at its lateral end (remaining with seven holes) for proper fitting. This adjustment was likely due to the medially located fracture line (Figure 1).

The SC joint subluxation, while rare, is typically treated non-surgically [8,10]. Closed reduction is usually achieved by addressing the associated injuries and mobilizing the affected shoulder. Thus, we proceeded with the treatment of the fracture and acromioclavicular (AC) dislocation as presented. The reduction of the SC joint was assessed with postoperative computed tomography, which demonstrated a satisfactory reduction of the subluxation. Immobilization with a sling was then maintained for two weeks as part of the treatment for the SC subluxation [10].

## Conclusions

There has been no need for reoperation to date, which is an additional benefit of the techniques employed. The patient has shown satisfactory progress in the nine months of follow-up, remaining pain-free, with a full range of motion and no aesthetic complaints, and has returned to her pre-injury level of activity, expressing satisfaction with the treatment. She was informed about the present study and provided a signed informed consent form (ICF).

The true incidence of the clavicle triple injury remains uncertain, and new reports may emerge with heightened clinical suspicion and the use of diagnostic tomographic methods. We aim to contribute to future studies on this exceedingly rare triple injury of the clavicle while presenting a therapeutic option for cases of acromioclavicular dislocation associated with midclavicular fractures.
